# Neuromodulation of OCD: A review of invasive and non-invasive methods

**DOI:** 10.3389/fneur.2022.909264

**Published:** 2022-08-09

**Authors:** Alexandra Kammen, Jonathon Cavaleri, Jordan Lam, Adam C. Frank, Xenos Mason, Wooseong Choi, Marisa Penn, Kaevon Brasfield, Barbara Van Noppen, Stuart B. Murray, Darrin Jason Lee

**Affiliations:** ^1^Department of Neurosurgery, Keck School of Medicine, University of Southern California, Los Angeles, CA, United States; ^2^Department of Neurosurgery, University of Michigan, Ann Arbor, MI, United States; ^3^Department of Psychiatry, Keck School of Medicine, University of Southern California, Los Angeles, CA, United States; ^4^Department of Neurology, Keck School of Medicine, University of Southern California, Los Angeles, CA, United States; ^5^Keck School of Medicine, University of Southern California, Los Angeles, CA, United States

**Keywords:** neuromodulation, obsessive compulsive disorder (OCD), deep brain stimulation (DBS), TMS, VNS

## Abstract

Early research into neural correlates of obsessive compulsive disorder (OCD) has focused on individual components, several network-based models have emerged from more recent data on dysfunction within brain networks, including the the lateral orbitofrontal cortex (lOFC)-ventromedial caudate, limbic, salience, and default mode networks. Moreover, the interplay between multiple brain networks has been increasingly recognized. As the understanding of the neural circuitry underlying the pathophysiology of OCD continues to evolve, so will too our ability to specifically target these networks using invasive and noninvasive methods. This review discusses the rationale for and theory behind neuromodulation in the treatment of OCD.

## Introduction

Obsessive-compulsive disorder (OCD) is a disabling psychiatric condition that affects ~2% of the population. The disorder is characterized by the presence of obsessions, compulsions, or both, which are time consuming (>1 h/day) and cause significant distress and impairment in important areas of functioning (1). Obsessions are persistent, unwanted thoughts or images that cause distress and anxiety, and compulsions are repetitive behaviors or mental rituals that are aimed to neutralize the distress and anxiety. First line treatments for OCD are cognitive behavioral therapy (CBT) with exposure and response prevention (ERP) and selective serotonin reuptake inhibitors (SSRIs). Additional pharmacotherapy includes the tricyclic antidepressant (TCA) clomipramine as well as augmentation with neuroleptics. Even among those treated with these evidence-based psycho- and pharmacotherapies, approximately one third of patients have refractory disease with continued disabling symptoms. Given this ongoing and significant disease burden, optimized and novel therapies are needed. Toward this end, various neuromodulation approaches, such as transcranial magnetic stimulation (TMS), transcranial direct current stimulation (tDCS), transcranial alternating current stimulation (tACS), and deep brain stimulation (DBS), have shown efficacy in improving OCD symptom burden in this otherwise treatment-refractory population. This review discusses the rationale for and theory behind neuromodulation in the treatment of OCD.

## The neurobiology of OCD: Circuit dysfunction

It is important to underscore that all novel neuromodulatory treatments must be firmly rooted in hypotheses relating to the neurobiological underpinnings of OCD. While early research into OCD focused largely on psychological mechanisms, the development and evolution of modern neuroimaging techniques has advanced our understanding of its neurophysiological underpinnings. Structural techniques such as voxel-based morphometry, surface-based cortical thickness, and tractography allow for the study of gray matter volume and integrity of white matter tracts. Functional imaging techniques such as positron emission topography (PET) and functional magnetic resonance imaging (fMRI) provide indirect measurements of brain activity in regions of interest and functional connectivity across nodes within and between brain networks. Direct methods of measuring brain activity include single unit recording, local field potentials, electrocorticography, and electroencephalography, the latter also being able to measure connectivity between cortical regions in particular frequency bands. Combined, these techniques have contributed greatly to our understanding of circuit dysfunction in OCD.

Current theories of OCD pathophysiology posit dysfunction in several distinct, yet overlapping brain circuits that involve connections from cortex to basal ganglia to thalamus and reciprocally back to cortex, known as cortico-striato-thalamo-cortical loops (CSTC) (2, 3). A number of distinct networks within this pathway were described by Alexander et al. (4). based on anatomophysiological findings and have subsequently been validated based on functional connectivity maps (4–6). However, the number of distinct networks has varied among descriptions, and the degree of topographical segregation between circuits remains an area of active research. Several CSTC circuits have been implicated in OCD based on clinical symptom clusters (i.e., obsessions about contamination compared to obsessions about symmetry), structural and functional neuroimaging, results from animal models, and patient response to treatment. Here we provide an overview of this evidence as it pertains to CSTC dysfunction in OCD.

## Lateral orbitofrontal-ventromedial caudate pathway

One of the earliest circuits implicated in OCD is the lateral orbitofrontal-ventromedial caudate pathway. As described by Alexander et al. (4), neurons in the lateral orbitofrontal cortex (lOFC) project to the ventromedial caudate, dorsomedial globus pallidus internus (GPi), and ventral anterior thalamus before projecting back to the lOFC (4). The role of the lOFC in behavior adaption and reversal learning is well-established (7–9). It has been postulated that dysfunction within the lOFC-ventromedial caudate circuits underlie persistence of habit-driven over goal-directed behavior (10). Similarly, the caudate is known to be involved in learning and goal-directed behavior, including a critical role in habit formation, and is thought to be hyperactive in OCD (11). These hypotheses are supported by studies showing increased gray matter volume, metabolism/activation (as measured by PET and fMRI), and functional connectivity in the lOFC, caudate and lOFC-striatal pathway, correlated with symptoms (12–15). Work from animal studies has demonstrated increased single-unit activity in the IOFC in pharmacological models of OCD, and OCD-like symptoms in mice after optogenetic stimulation of OFC-ventromedial striatum (16, 17). Interestingly, successful pharmacological or psychological treatment of OCD leads to normalization of OFC and caudate nucleus activity, again supporting a hypothesis that these structures are involved in the pathogenesis of OCD symptoms (18).

## Limbic network

**Figure 1:**
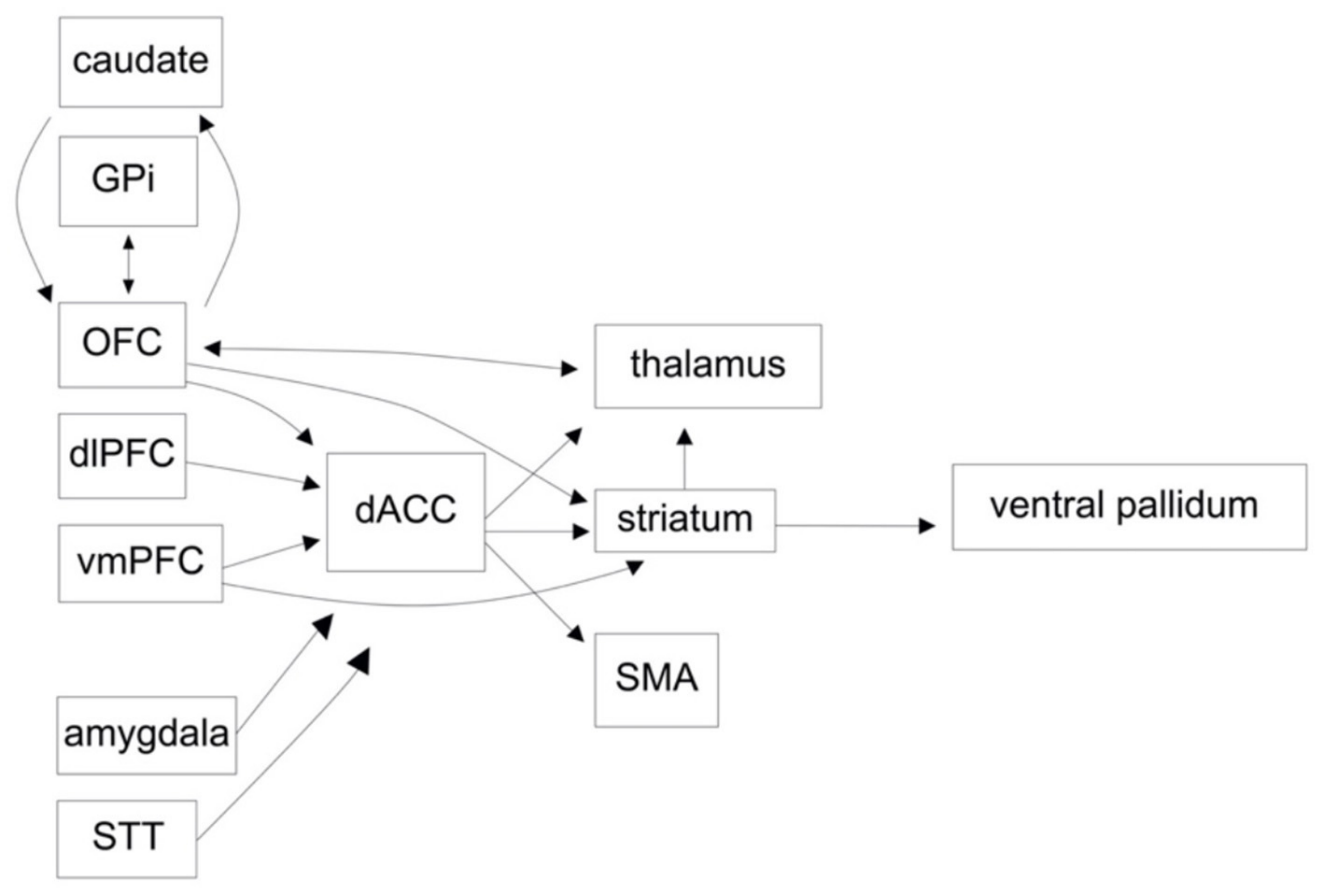
A functional circuit diagram depicting relevant afferent and efferent connectivity in the cortico-striatal-cortical and orbitofrontal circuits, implicated in the pathogenesis of obsessive compulsive disorder (OCD).

The limbic network is principally involved in emotion, reward-motivated behavior, learning, and memory. The structures considered part of the limbic network have been updated over time as novel evidence suggests roles for various brain regions (19). Generally accepted components include the hippocampus, entorhinal cortex, fornix, mammillary bodies, anterior nucleus of the thalamus (ANT), anterior cingulate cortex (ACC), medial prefrontal cortex, amygdala, septal nuclei, nucleus accumbens (NAcc), and hypothalamus (20, 21). In terms of a CSTC loop, the ventral striatum receives inputs from the medial OFC (mOFC), ventromedial prefrontal cortex (vmPFC) and the dorsal anterior cingulate cortex (dACC), and in turn projects to the ventral pallidum and mediodorsal nucleus of the thalamus (Figure 1). These structures are heavily regulated by dopaminergic input, which serves as an expectation/outcome error signaling mechanism to facilitate learning and memory. Dysregulation of the limbic system has been implicated in a number of models of OCD pathogenesis, including increased classical conditioning of compulsions; reduced modulation of a frontal-based habit system by hippocampal-based memory; and a deficit of implicit learning with compensation by explicit learning (22–24). Indeed, patients with OCD show reduced task-based reward learning, correlated with reduced activity in, and connectivity between, limbic structures such as the NAcc, ventral putamen, vmPFC, OFC, hippocampus, and amygdala (15, 24–27). During episodes of increased symptomatology there is hyperactivation in limbic structures (28, 29). There is a discrepancy in resting state functional connectivity between medicated and unmedicated OCD patients, with increased connectivity found in medicated OCD patients, and reduced connectivity, both within the limbic system and in other networks, in unmedicated patients (30, 31). While the latter may reflect deficits in reward processing and learning, there may be measurable changes in network connectivity due to medication. Further supporting this hypothesis, serotonin-selective reuptake inhibitors (SSRIs) have been shown to increase frontostriatal connectivity in patients with depression and pediatric patients with OCD (32, 33).

## Default mode network

Termed the “task-negative” network, the default mode network is involved in internal mentation. In early studies using PET, decreased activity of the medial prefrontal cortex, lateral parietal cortex, posterior cingulate cortex (PCC), and precuneus was observed during externally orientated tasks with subsequent increased activity at rest, defining this network (34). However, a number of other structures, such as the medial temporal lobe, have been implicated in internal mentation, and it has been proposed that the default mode network may be comprised of multiple related networks (35). The default mode network has been the object of study in a number of neurocognitive and psychiatric conditions such as major depressive disorder and Alzheimer's disease. In OCD, dysfunction in the default mode network is thought to underlie increased self-referential attention to persistent thoughts. Consistent with this, individuals with OCD have been found to have increased task-based functional connectivity in the default mode network (36). Decreased PCC activation and increased PCC-vmPFC functional connectivity were found in OCD patients during reward processing (37). This finding corroborates evidence of reward processing deficits and limbic network dysfunction, while also suggesting a shift of attention away from external rewards toward internal thoughts through an inability to deactivate the default mode network. While findings vary between studies, meta-analysis of resting-state functional connectivity data has shown aberrant connectivity within the default mode network, and hypoconnectivity with the frontoparietal and salience networks, suggesting an abnormal balance between self-referential and task-related attention (38). After SSRI therapy, a normalization of default mode network hypoconnectivity with other “task-positive” networks, e.g., the dorsal attention network, has been demonstrated (39).

## Salience network

Although involved in a wide range of functions, the salience network has been particularly implicated in attention switching, response, and response inhibition. Specifically, it has been postulated to regulate the transition between the task-based frontoparietal executive control network and task-negative default mode network. Also referred to as the cinguloopercular, cinguloinsular or ventral attention network, the salience network consists of two principal structures, the dACC and anterior insula, in addition to a CSTC loop containing the dlPFC, dorsolateral caudate, and mediodorsal nucleus of the thalamus (4, 5, 40). Modulation of activity in these areas has been shown during tasks involving salient stimuli, error awareness, and uncertainty (41–43). Consistent with the role of the salience network and its components, it has been proposed that dysfunction of this network can lead to aberrant processing of stimuli, attentional control, physiological reactions, and motor response (44). In patients with OCD, there is increased activity in the anterior insula, dorsal caudate, and dACC in task-based protocols, with this activity positively correlating with symptoms (32, 45–47). In rodent models of OCD, lesioning of the anterior insula decreases compulsivity (48). Furthermore, meta-analysis of resting-state functional connectivity in OCD has demonstrated hypoconnectivity not only within the salience network, but between the salience network, default mode network, and frontoparietal networks (38). Consistent with the interplay between these three networks, in OCD there is thought to be aberrant processing of salient stimuli and an inability to shift attention away from internal obsessions mediated by the default mode network toward goal-directed behavior mediated by the frontoparietal network (44).

## Methods of neuromodulation

### A basis for neuromodulation

Long before advanced imaging studies and animal studies helped to elucidate the neural circuitry involved in OCD, clinicians have sought to provide brain-based treatments for this disorder. Early strategies implementing prefrontal lobotomies and leukotomies showed some promise in alleviating symptoms; however, the side effects were significant (49). With the dawn of stereotactic surgery, more specific lesioning strategies such as cingulotomies and anterior capsulotomies showed more promising results with more tolerable side effects (50). As more effective pharmacotherapies became available with even fewer adverse effects than the more precise surgical techniques, the interest in surgical management waned.

**Figure 2:**
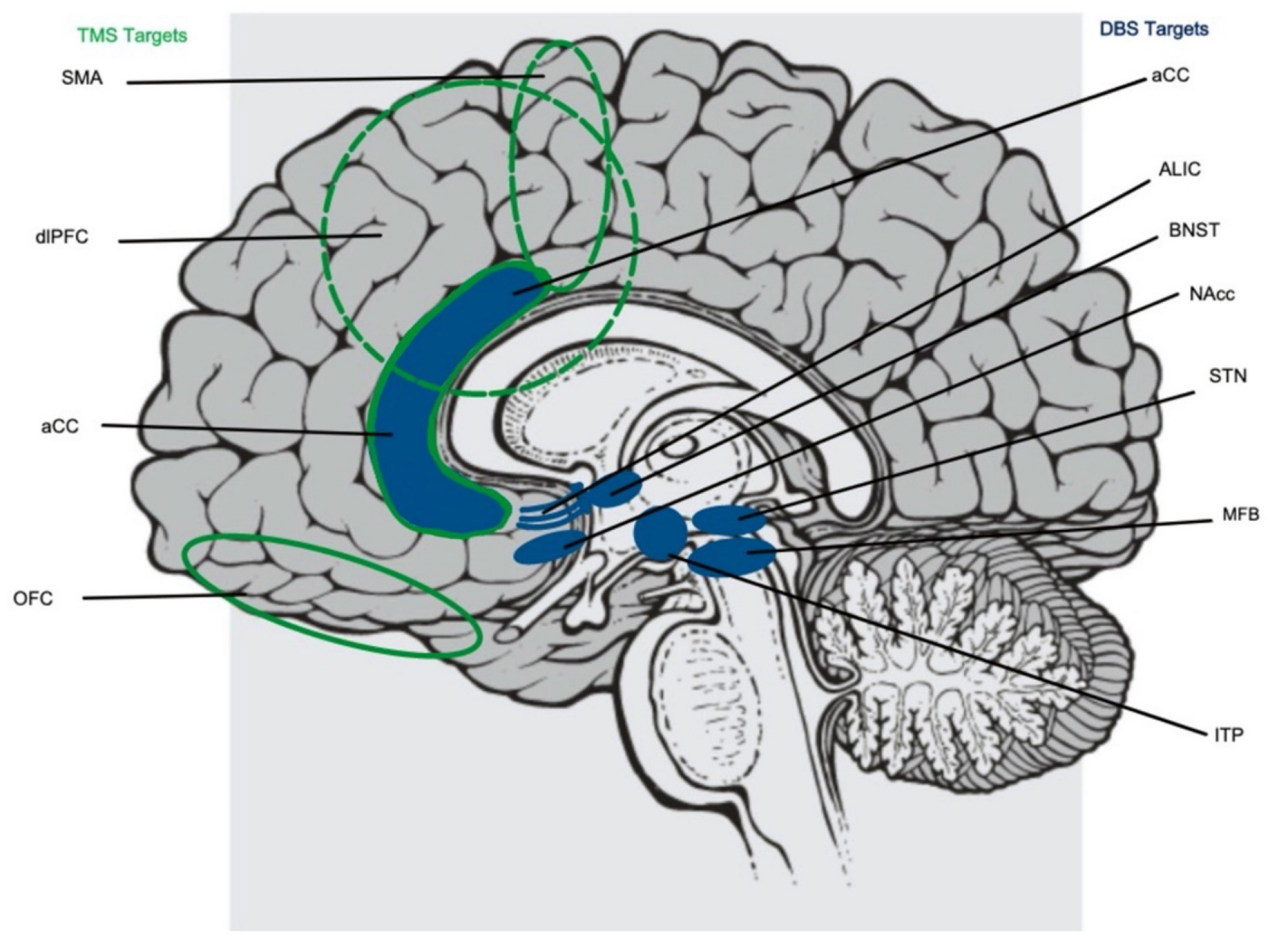
Targets for both transcranial magnetic stimulation (TMS, highlighted in green to the left) and deep brain stimulation (DBS, highlighted in blue to the right) implicated in the treatment of obsessive compulsive disorder. TMS targets include the supplementary motor area (SMA), dorsolateral prefrontal cortex (dlPFC), and orbitofrontal cortex (OFC). DBS targets include the anterior limb of the internal capsule (ALIC), basal nucleus of the stria terminalis (BNST), nucleus accumbens (NAcc), subthalamic nucleus (STN), medial forebrain bundle (MFB), and inferior thalamic peduncle (ITP). The anterior cingulate cortex (aCC) is a target of both TMS and DBS.

Around this same time, electroconvulsive therapy (ECT) also emerged as a less invasive strategy in the treatment of a variety of psychologic disorders. The results for ECT in OCD have thus far been inconsistent with some studies showing benefit while others showed no improvement, but this strategy identified a clear role of applying external fields to the brain in order to influence neural networks (51). The lessons learned from early surgical lesioning and applying electric fields to the brain were combined with emerging work in understanding the neurologic aberrations in OCD, laying the groundwork for more specific neuromodulatory strategies like transcranial magnetic stimulation (TMS), transcranial direct current stimulation (tDCS), transcranial alternating current stimulation (tACS), and deep brain stimulation (DBS) (Table 1, Figure 2).

**Table 1:** Summary of neuromodulatory modalities in the treatment of OCD.

	**Non-invasive**			**Invasive**		
Modality>	Transcranial direct current stimulation (tDCS)>	Transcranial alternating curent stimulation (tACS)>	Transcranial magnetic stimulation (TMS)>	Deep brain stimulation (DBS)>	Vagal nerve stimulation (VNS)>	Surgical lesioning>
Targets>	pre-SMA/SMA, mPFC, DLPFC, OFC>	DLPFC, mOFC>	DLPFC, SMA, OFC, mPFC, ACC>	ALIC, VC/VS, NAcc, STN, ITP, BNST>	Vagus Nerve>	Limbic leucotomy, subcaudate tractotomy, anterior capsulotomy>
Procedure>	5–20 sessions, 1–2 sessions per day, (20 min stimulation)>	8–20 sessions, 3 sessions/week, (20 min)>	10–30 sessions, 5 sessions/week>	Lead implantation, generator implantation, follow up for parameter optimization>	VNS implantation>	Surgery for lesioning>
Significant adverse effects>	None>	None>	Rare, mood disturbances>	Hemorrhage, infection, suicdaility, impulsiveness, mood disturbance>	Infection, cough, dysphagia, voice change>	Hemorrhage, infection, suicdaility, impulsiveness, mood disturbance>

*SMA, supplementary motor area; mPFC, medial prefrontal cortex; DLPFC, dorsolateral prefrontal cortex; mOFC, medial orbitofrontal cortex; ACC, anterior cingulate cortex; ALIC, anterior limb of the internal capsule; VC/VS, vental capsule/ventral striatum; NAcc, nucleus accumbens; ITP, inferior thalamic peduncle; BNST, bed nucleus of the stria terminalis*.

### Transcranial magnetic stimulation

TMS is a non-invasive neuromodulation technique that has shown efficacy in the treatment of a variety of neurologic and psychiatric conditions and has FDA-approval for treatment-refractory major depressive disorder, anxiety associated with depression, treatment-refractory OCD, smoking cessation, and migraine with aura (52). Mechanistically, a brief but strong magnetic field is generated by transiently passing electricity through wire windings of various configurations contained within the stimulation coil, which is placed against the head. This magnetic field passes through the scalp and skull to induce an electric field within the underlying brain region, causing depolarization of neurons. This process is repeated at varying frequencies, and in this way, TMS can alter electrical activity within the targeted brain region.

A variety of stimulation protocols have been studied, though they generally fall into three groups: high frequency, low frequency, and theta burst. High frequency stimulation (pulse repetition rates between 5 and 20 Hz) increases cortical excitability while low frequency stimulation (1 Hz) tends to decrease cortical excitability (52–54). Similarly, it is hypothesized that high frequency TMS influences behavioral change through long-term potentiation (LTP) while low frequency TMS initiates long-term depression (LTD) (55, 56). Through these mechanisms, the effects of stimulation on cortical excitability persist beyond the duration of the treatment. A recently introduced stimulation pattern known as theta burst stimulation (TBS), first described in human motor cortex by Huang et al. (57), delivers pulses in a theta frequency band (3 pulses at 50 Hz repeated at 5 Hz) to mimic endogenous brain rhythms. TBS can also induce either excitatory effects—if delivered intermittently (intermittent TBS, iTBS)—or inhibitory effects—if delivered continuously (continuous TBS, cTBS)—and allows for shorter treatment times and lower treatment intensities, which are generally better tolerated by patients.

As discussed previously, there are abnormalities in signaling in multiple anatomic structures and neural networks in OCD, and these pathways provide a variety of targets for TMS. There have been a number of randomized control trials that have investigated the effectiveness of TMS in treating OCD by targeting the DLPFC (58–70), the SMA (71–77), the OFC (78, 79), and the mPFC (80, 81). However, there is notable heterogeneity in these studies in terms of number of participants, stimulation frequency, number of treatments, length of follow-up, and efficacy in reducing OCD symptom burden. Several recent systematic reviews and meta-analyses have been conducted to assess the overall evidence for the use of TMS in treating OCD (82–87). The meta-analysis by Liang et al. found that targeting the right DLPFC with low frequency stimulation had the greatest effect size, while low frequency stimulation of the SMA and high frequency stimulation of the DLPFC were also more effective than sham (86). Notably, the authors find the overall quality of this evidence to be very low to low, based on the GRADE framework. These results align with recent treatment guidelines which suggest “possible efficacy of low frequency rTMS of the right DLPFC” (52). The meta-analysis by Perera et al., which included 26 total studies, demonstrated that targeting of the bilateral DLPFC with either high or low frequency stimulation has the greatest effect size (87). While these meta-analyses and treatment guidelines generally indicate the DLPFC as the preferred target for rTMS in OCD, in a large sham-controlled randomized trial, Carmi et al. (81) found significant improvement in OCD symptoms as measured by the Yale-Brown Obsessive Compulsive Scale (Y-BOCS) with high frequency, deep rTMS of the mPFC (81, 88). Based on the results of this trial, the FDA has approved TMS for treatment-refractory OCD.

Several questions have arisen from these prior studies. Perhaps most prominent is which cortical region should be targeted for an individual patient, given the heterogeneity of OCD symptoms, neural circuit dysfunction, degree of symptom burden, and presence of co-morbid psychiatric disorders (89). Imaging studies have shown that distinct neural circuits are differentially activated in patients with different OCD symptoms (90). One approach that may improve treatment outcomes is the selection of the cortical stimulation target based on personalized symptom profile, neuroanatomy, and/or circuit dysfunction. Similar approaches have shown promise for improving treatment outcomes in TMS for depression and DBS for OCD (91–93).

Another important question in the administration of TMS is whether brain state during stimulation has implications for treatment outcome. Few studies have examined this question and most current clinical use of TMS is independent of brain state, with patients permitted to engage in any variety of tasks during treatment (watching movies, listening to music, utilizing cell phones, etc.). Broadly, state-based TMS has been examined in a variety of conditions and generally been shown to be effective: in bulimia (94) food cues were presented prior to stimulation; in PTSD (95) trauma cues were presented prior to stimulation; in nicotine use disorder (96) smoking cues were presented prior to stimulation; and in depression (97) stimulation was coupled with CBT. Within OCD, two RCT's have addressed state-dependence by utilizing symptom-provocation immediately prior to TMS treatment; both studies and found that HF mPFC stimulation coupled with provocation led to an improvement in OCD symptom burden (80, 81). Overall, this is an important avenue of future research and various stimulation locations in OCD may show effectiveness if coupled with brain circuit activation prior to or during stimulation.

Taken together, there is evidence from a number of studies that TMS applied to a variety of cortical sites, either with or without symptom-provocation, can improve OCD symptom burden. Future studies should examine such outstanding questions as: (1) Might different cortical stimulation targets improve specific OCD symptoms (e.g., harm-based vs. non harm-based obsessions); (2) does underlying patient-specific circuit dysfunction impact efficacy of cortical stimulation targets; and (3) does concurrent brain state (governed by symptom-provocation or engagement in psychotherapy) impact TMS efficacy. Other future studies utilizing TMS in OCD should seek to gain a better understanding of the pathophysiologic mechanisms in order to design more specific neuromodulatory strategies. A good example of how this may be accomplished comes from the study of Alzheimer's Disease (AD). Di Lorenzo and colleagues investigated spike timing dependent cortico-cortical plasticity (STDP) in AD patients by applying timed stimulation to the posterior parietal cortex then the primary motor cortex or vice versa and studying motor-evoked potentials (98). With this study, they were able to determine that STDP was inhibited in AD patients when compared to healthy controls. This study highlights the role of TMS as a tool to aid in our understanding of networks and the pathophysiologic mechanisms underlying disease. Similar experiments with TMS could be applied in the study of OCD.

### Transcranial direct current stimulation/Transcranial alternating current stimulation

tDCS is another non-invasive neuromodulation strategy that has been studied in the treatment of OCD. In delivering tDCS, a weak direct current is conducted across the brain between two electrodes (99). Cathodal stimulation is thought to be inhibitory while anodal stimulation is thought to have excitatory effects (99). Typical stimulation parameters used in the literature are 1–2 mA delivered for 20–30 min, and patients typically undergo repeat treatments for 10–20 treatments. Numerous studies have investigated the effects of tDCS in the treatment of refractory OCD. The targets selected were the same as those used for TMS, including pre-SMA/SMA, mPFC, DLPFC, and OFC (84).

There have been several randomized control trials that have targeted the pre-SMA/SMA (100–102), the mPFC (103, 104), OFC (105). D'Urso et al. (100) found that cathodal but not anodal stimulation of the bilateral pre-SMA elicited improvement in OCD symptoms, while Gowda et al. (101) showed anodal stimulation resulted in improvement when compared to sham. Todder et al. found that tDCS to the mPFC showed a decrease in obsession-induced anxiety (103). In another study looking into tDCS to the mPFC, Adams et al. showed that anodal stimulation showed significant improvement in therapeutic safety learning. Interestingly using functional imaging, they showed that functional connectivity increased between the frontal pole and the middle and superior frontal gyri, while they found that the functional connectivity between the insula and the basal ganglia decreased as did the functional connectivity between the DMN and salience networks (104). Bation et al. showed that left anodal stimulation of the left OFC had short term effects but failed to show significant improvement during a 3 month follow up (105). Overall, these studies all showed the feasibility and efficacy of tDCS in targeting multiple brain regions. Larger randomized control trials are needed to define the optimal target and treatment parameters, but tDCS is an efficacious, non-invasive neuromodulatory modality that extends the armamentarium of treatment for refractory OCD.

Another modality that has shown some promise in the treatment of refractory OCD is tACS. tACS uses sine wave stimulation at the desired frequency range. tACS protocols employ much less stimulation intensity than tDCS (0.6 mA vs. 2 mA) (106). There have been few studies into using tACS for the treatment of OCD. In a small case series, Klimke et al. showed that tACS to the bilateral DLPFC was effective in improving OCD symptoms in 6 out of 7 patients with an average decrease in the Y-BOCS score by 52% (107). In a recent study by Grover et al. using high definition tACS to the mOFC, they found that patient-specific beta-gamma modulation influences reward- but not punishment-based learing and behaviors when compared to sham, and these protocols were found to decrease obsessive-compulsive symptoms in patients with sub-clinical obsessive compulsive symptomatology (108). The early results in tACS for OCD have shown feasibility and promise, but more work is needed to optimize this modality.

### Invasive neuromodulation

The 1930s saw the expansion of psychosurgery with prefrontal leucotomies and, after the development of the stereotactic frame in the 1940s, more targeted lesioning, including capsulotomy, cingulotomy, and subcaudate tractotomy came into favor. These destructive techniques aim to downregulate activity in individual structures or neural pathways that have been implicated in the neurocircuitry of OCD. While anterior capsulotomy and cingulotomy are still used today, ablative techniques have largely been replaced by neuromodulation, including deep brain stimulation (DBS) and vagus nerve stimulation (VNS). Unlike ablative procedures, neuromodulation is reversible and modifiable. As the only Food and Drug Administration (FDA)-approved form of invasive neuromodulation for OCD, DBS utilizes pulsed electricity at a specified pulse width, voltage, and frequency to modulate the activity of deep target structures (109–111).

As the pathogenesis of OCD becomes better understood as being due to dysfunctional brain circuitry, the regions targeted with DBS have evolved to specifically influence the neural pathways comprising these networks (112). The most common targets of DBS for OCD include the anterior limb of the internal capsule (ALIC), nucleus accumbens (NAcc), bed nucleus of the stria terminalis (BNST), subthalamic nucleus (STN), and inferior thalamic peduncle (ITP). Treatment response is most frequently evaluated with the percent decrease of Y-BOCS, with secondary outcome measures including the Hamilton Depression Rating Scale (HAM-D) and (Hamilton Anxiety Ranting Scale (HAM-A) scores (113, 114). When using Y-BOCS to evaluate treatment response, a cutoff of 35% decrease of symptoms is typically used to classify subjects as responders vs. non-responders (115).

### ALIC

The anterior limb of the internal capsule (ALIC) connects subcortical nuclei with the prefrontal cortex (116). It has been implicated in numerous psychiatric disorders and is targeted with lesioning surgery via anterior capsulotomy as well as modulation by DBS. The therapeutic mechanism of these modalities is likely through interrupting the fibers traversing the ALIC and thus decreasing overactivity of the thalamocortical circuits thought to underlie OCD pathophysiology (117). The first study of DBS of the ALIC by Nuttin et al. was performed based on known targets of capsulotomy (118). In this non-blinded interventional cohort study, four patients received bilateral stimulation of the ALIC in locations per prior ablative protocols. Three of the four patients improved. Since then, there have been several studies demonstrating the effectiveness of ALIC stimulation for OCD (119–122).

A recent study of 70 patients by Denys et al. demonstrated effectiveness of ventral ALIC (vALIC) stimulation for OCD (121). This non-blinded cohort study showed an average Y-BOCS decrease of 40% with 62% of subjects being classified as responders. Within the responder group there was a 62% decrease of OCD symptoms as measured by Y-BOCS. The authors found that Y-BOCS decreased initially with standardized stimulation, then continued to decrease over the following year. The improvement in symptom reduction over time may have been due to optimization and personalization of stimulation parameters. The subjects' HAM-A and HAM-D scores decreased by 55 and 54%, respectively. The reduction in HAM-A and HAM-D scores was stable throughout the study, that is, the scores did not continue to decrease over the following year. The study was limited to 1 year and thus did not evaluate long-term results of ALIC DBS for OCD.

Within the ALIC, there have been investigations of whether individual fiber bundles could be targeted for optimal benefit. Indeed, Liebrand et al. found that targeted stimulation of the ALIC in closer proximity to the medial forebrain bundle (MFB) (rather than the anterior thalamic radiation), had a significant therapeutic benefit (123). Other groups have described methods to delineate and target individual tracts and predict treatment response, and the topic is still in debate, which will be discussed further (124–127). Additionally, several groups have noted clinical signs during awake lead placement and programming which are associated with better outcomes and may reflect proximity to specific fiber bundles. The most common of these is the “mirth response,” in which patients are noted to smile and laugh during lead placement and stimulation (128, 129).

Despite good response and remission rates of ALIC stimulation, its clinical usefulness is somewhat constrained by the high stimulation intensity required for beneficial results (84, 119). Most clinical studies show effectiveness with ALIC stimulation only after extensive programming and with amplitudes of 5–10 V. Compared to DBS for movement disorders (Parkinson's disease, essential tremor, dystonia), amplitudes at this intensity necessitate frequent replacement of the implantable-pulse-generator.

Adverse effects of ALIC stimulation include hypomania (including restlessness, agitation, and impulsivity). Denys et al. found that these symptoms were often correlated with stimulation changes and were typically transient (121). Other adverse effects include headaches, anxiety, and rarely, seizures (122). Additionally, some patients report transient stimulation-induced facial muscle contraction (119).

### NAcc and VC/VS

The nucleus accumbens (NAcc), known to play a significant role in reward behavior, is located in the basal forebrain ventral to the ALIC and rostral to the anterior commissure. The NAcc makes up the ventral striatum with the olfactory tubercle. The ventral capsule ventral striatum (VC/VS) consists of the ventral portion of the anterior limb of the internal capsule as well as the adjacent ventral striatum. Its use as a DBS target was based on the hypothesis that OCD is due to a dysfunctional reward pathway in the context of successful anterior capsulotomy results for the treatment of OCD (24, 130). DBS targeting the NAcc reduces excessive connectivity between the NAcc and the prefrontal cortex, as measured by EEG and fMRI (110).

Distinct regions in the NAcc may play separate functional roles, as they receive input from different areas in the brain. The medial aspect and the shell of the NAcc receive input from the rostral part of the anterior cingulate cortex (131). This innervation occurs through multiple white matter tracts, including the amygdalofugal tract between the NAcc and anterior SCG. This tract originates from the basolateral and central nuclei of the amygdala before passing under the lentiform nucleus. It then runs medially alongside and underneath the anterior commissure and internal capsule before dividing into ascending and descending branches. The ascending branch of the amygdalofugal tract courses through the NAcc, then to the anterior SCG, before terminating in the septal nuclei.

There have been several successful trials of DBS of the NAcc and VC/VS for the treatment of OCD (120, 132–134). Sturm et al. performed unilateral and bilateral high-frequency stimulation of the NAcc in four patients (132). In this study, DBS electrodes were placed at the junction of the ventral internal capsule (VIC) with the NAcc. Their group found that bilateral stimulation did not show more OCD symptom reduction compared to unilateral stimulation of the right NAcc in the first patient, so further patients were treated with unilateral stimulation of the right NAcc. They noted a significant reduction in OCD symptoms in three out of four patients studied.

Denys et al. performed double-blinded bilateral NAcc stimulation in sixteen patients (133). In this study, bilateral electrodes were placed in the NAcc following the angle of the ALIC. They found that Y-BOCS decreased by 52% after stimulation. Nine out of sixteen patients were considered responders, with a mean improvement in obsessive-compulsive symptoms of 72% within the group. Additionally, depressive and anxiety scores decreased by 55 and 57%, respectively, as measured by HAM-D and HAM-A scores. Of note, stimulation-induced elevated mood was seen in all patients in this study, even those who did not respond in terms of their OCD symptoms. In a multi-site clinical trial of 26 patients, Greenberg et al. also found significant reduction in co-morbid depression and anxiety after VC/VS stimulation (134). In this study, there was a 40 and 53% decrease in HAM-D and HAM-A scores, respectively.

There are several potential adverse effects and complications of NAcc and VC/VS stimulation, including mood disturbances (134). Mood elevation is most often seen with increased stimulation, with some subjects progressing to hypomania. Mood depression has been seen when stimulation is decreased or turned off. In patients with co-morbid depression, abrupt cessation of stimulation can lead to return to pre-DBS levels of depression. Additionally, while anxiety is often improved with stimulation, there have been reports of transiently increased anxiety.

### STN

The subthalamic nucleus (STN) is a common target for neuromodulation in Parkinson's Disease as it plays a role in both behavioral as well as motor control. The topographic localization of emotional vs. motor control has been demonstrated through stimulation of sub-regions of the STN, with the anteromedial portion being specifically implicated in emotional control (135). Because the STN is widely utilized as a target in deep brain stimulation for Parkinson's disease, its stimulation was somewhat incidentally found to have therapeutic effects for OCD management in patients undergoing DBS with co-morbid OCD and Parkinson's disease (136).

Mallet et al. examined bilateral stimulation of the anteromedial subthalamic nucleus (137). In this randomized, double-blinded study of eight patients, six out of the eight patients responded to STN stimulation as measuring by Y-BOCS improvement, with an average Y-BOCS decrease of 37% after the stimulation period vs. 7% after the sham period. Notably, this study found no improvement of depression or anxiety symptoms.

Tyagi et al. compared DBS of the STN with DBS of the VC/VS in a randomized, double-blinded study (138). They found that stimulation of the STN provided similar control of OCD symptoms with Y-BOC decreased 45% in STN compared to 53% in VC/VS. The two targets differed in secondary effects, however. STN stimulation was associated with improved cognitive flexibility as measured by the Cambridge Neuropsychological Test Automated Battery Intra-Extra Dimensional Set-Shift (IED) task, and VC/VS stimulation was not. VC/VS stimulation, on the other hand, demonstrated improved mood symptoms compared to STN stimulation, as measured by the Montgomery-Asberg Depression Rating Scale (MADRS).

One specific drawback of STN stimulation is the comparatively long duration of treatment required to reach the full effect, which can be on the order of years rather than weeks or months for other targets (139). This was outlined in case study by Wojtecki et al. in which full results were not seen until 3 years of stimulation. Adverse motor effects of STN-DBS for OCD can include hyperkinetic movement disorders such as choreiform dyskinesias.

### BNST

The bed nucleus of the stria terminalis (BNST) is located in the basal forebrain caudal to the anterior commissure and inferomedial to the ALIC (140). It connects to the greater limbic circuitry via the stria terminalis, which wraps around the deep brain structures to connect the BNST to the amygdala. It has been implicated in numerous physiologic processes such as fear, anxiety, and goal-directed behavior. Its use as a target for stimulation was proposed after observing the effects of ALIC stimulation (141).

The first study of BNST stimulation for OCD, a long-term clinical trial of 24 patients published by Luyten et al. showed a significant improvement in Y-BOCS, HAM-D, and HAM-A scores (141). Additionally, this study demonstrated significantly greater effect size when compared with ALIC stimulation. In a further longitudinal study of the same patient cohort, Raymaekers et al. found long-term sustained effects of DBS of the BNST for OCD and co-morbid depression and anxiety (142). A randomized, double-blind, sham-controlled study of bilateral stimulation of the BNST was recently completed by Mosley et al. (143). This study saw a significant reduction of Y-BOCS during the blinded phase, with a mean difference of 4.9 points (15%) comparing on vs. sham. After the blinded phase, the open-label portion of the study saw further improvement at the 1-year mark, with a mean reduction of 17.4 points (47% improvement compared to pre-DBS). The discrepancy between blinded and open-label results is multi-factorial but likely due to a relatively short blinded portion, which took place prior to the open-label portion, with extensive programming optimization taking place during the open-label portion.

Adverse effects of BNST stimulation are generally similar to those of other DBS targets in the basal forebrain, e.g., mood disturbances and agitation. Mosely et al. found two serious adverse events related to DBS device placement, but there were no serious psychiatric events in any patients. However, Luyten et al. noted several subjects with post-implantation seizures leading to study discontinuation (141). These seizures were not confirmed with EEG. The mechanism by which stimulation of the BNST may lead to seizures remains unclear.

### ITP

The inferior thalamic peduncle is a white matter pathway that travels bidirectionally from the thalamus to the orbitofrontal cortex and connects to the reticular activating system (144, 145). Its use as a target for stimulation for OCD was proposed after considering its prior role in subcaudate tractotomy for OCD control where ITP fibers were lesioned (146).

Studies of ITP stimulation for OCD control have shown significant benefit (144, 145). A preliminary clinical study of five patients by Jimenez et al. showed improved Y-BOCs of 51% (from 35 to 17.8), with these results sustained at 1-year follow up. A phase 1 open-label pilot study of five subjects by Lee et al. demonstrated a good safety profile with average Y-BOCS improvement of 52% across five patients. This clinical improvement was correlated with improvement of metabolic dysfunction in other implicated brain areas, including the right caudate and putamen, right SMA, right cingulum, bilateral motor areas, left temporal pole and left OFC, suggesting extensive metabolic changes associated with ITP stimulation. This study also noted a trend toward co-morbid depression improvement (*p* = 0.07) but was limited by small sample size.

Like ALIC DBS, ITP stimulation requires high stimulation amplitudes, necessitating frequent battery changes. Additionally, further study is needed to determine whether ITP stimulation improves co-morbid depression and anxiety in OCD patients.

### Defining networks

Obtaining a more accurate understanding of the functional networks governing OCD pathophysiology is crucial to identify the optimal targets. Connectomic analyses have sought to define the functional networks and predict treatment response by targeting components of these networks. Balderman et al. found that the connectivity between DBS electrodes and medial and lateral prefrontal cortices strongly predicted outcomes (124, 127). Another connectomic analysis by Li et al. sought to define a network that could explain why targeting the ALIC and the STN both alleviated OCD symptoms, and they found strong connectivity in a bundle that connects the dorsal anterior cingulate and ventrolateral prefrontal cortices to the anteromedial STN, indicating the involvement in this tract (125). Building upon this work, Bouwens van der Vlis et al. sought to find a unifying connectomic target for DBS by correlating outcomes data with tractography data in patients undergoing VC/VS DBS, and they found that the tract that was associated with best outcomes was a subpart of the ALIC that connects the prefrontal cortex with the STN and mediodorsal nucleus of the thalamus (147). In contrast to the other studies, Widge et al. used patient-specific connectomics and statistical modeling in an attempt to predict treatment response, but they found that no statistical model could predict response (148). There is a clear need for more studies into defining the functional networks in OCD and using this information to inform patient-specific targeting.

### VNS

Vagus nerve stimulation (VNS) is an alternative modality of brain stimulation with FDA approval for treatment-resistant epilepsy and treatment-resistant depression (TRD). VNS typically involves placement of electrodes around the cervical portion of the left vagus nerve with stimulation delivered in an intermittent but chronic manner (149, 150). Through stimulation of this cranial nerve, VNS indirectly modulates the activity of brain networks and has the potential to induce changes in cortical connectivity and excitability (151). The use of VNS in psychiatry has largely been confined to TRD and its efficacy in other psychiatric disorders have been less widely studied. To our knowledge, only one pilot study investigated its potential impact on OCD (152). In this study, seven patients with a diagnosis of treatment-resistant OCD received VNS for a period of 12 weeks. Three of these patients responded acutely during the 12-week period, and two patients demonstrated continued reduction in disease severity for 4 years after implantation. These results warrant further investigation of the efficacy of VNS as an adjunctive treatment for OCD in a larger sample size of participants.

## Discussion

As discussed in this review, there are multiple promising modalities to provide neuromodulation for the treatment of refractory OCD both non-invasively and invasively. DBS is of particular interest because it works to directly modulate the known networks continuously over longer timescales. One of the major limitations in DBS for OCD relates to the quality of evidence. In 2014, the Congress of Neurological Surgeons (CNS) and the American Society for Stereotactic and Function Neurosurgeons (ASSFN) released guidelines for DBS in OCD, and they found that there was only Level I evidence to support bilateral STN DBS over medical management in treatment-refractory OCD (153). The quality of evidence for other targets was level II, and there was inconclusive evidence to inform a best target. Despite considerable research in the field, there were no major updates to the 2020 CNS guidelines as there was no new Level I evidence (154). Similarly, the World Society for Stereotactic and Functional Neurosurgeons (WSSFN) published consensus guidelines on DBS for OCD (155). From their analysis of the literature, they determined that DBS to “ventral anterior capsule region (including bed nucleus of stria terminalis and nucleus accumbens) remains investigational. It represents an emerging, but not yet established therapy.” The guidelines from the two major societies of stereotactic and functional neurosurgeons indicate that there is a clear need for more, high quality evidence to better define targets and optimal parameters for DBS for OCD.

Another limitation in DBS for OCD is that many of the patients who would be candidates for DBS are not getting surgery. In 2009, the FDA granted a Humanitarian Device Exemption (HDE) for DBS to the ALIC in hopes of increasing access to this therapy. Unexpectedly, since the HDE was granted, the number of patients receiving DBS implantation has decreased (156). In a thoughtful analysis into the decline in DBS for OCD, Pinckard-Dover et al. identified the contributing factors (156). They found that at their own institution, there was approximately a 50% decline in DBS for OCD when comparing before and after the HDE was granted. They found that the main reason for this decline was the failure of private insurance companies to provide financial coverage for the procedure. Before the HDE was granted, the cost of the devices was largely covered in the NIH budgets for clinical trials, but afterwards, researchers expected that insurance companies would pick up these costs. This was not the case, and enrollment for clinical trials dwindled as a result. The future of providing this efficacious treatment to patients and the future of continued research will require more buy-in from private insurance companies.

Perhaps the principal issue that remains with respect to DBS is in defining the optimal targets. The targeted areas comprise overlapping regions, leads traverse multiple brain regions, and there is some variability in terms of the coordinates used in the literature. In one of the landmark studies, Greenberg et al. discussed targeting the VC/VS, and they found that better results were achieved with progressively more posterior target (134). This is thought to be because the distal contacts were in the NAcc or BNST. In their systemic review of DBS targets, Raviv et al. they found that there was considerable overlap in the striatal regions (ALIC, NAcc, VC/VS) with some studies using the same coordinates to describe distinct structures while some studies did not even report coordinates (157). They suggest a more careful anatomic consideration, consistency in reporting coordinates, and a change in nomenclature to “strial” vs. STN/pallidal/ITP. They posit that in order to improve OCD DBS outcomes, we must gain a better understanding of the subanatomy of the strial region and use patient-specific connectomic analysis to define the best individualized targets for the individual patient. To this end, in their extensive review of the relevant anatomy of the VC/VS and NAcc, Park et al. found that the caudal part of the NAcc passing through the IC-AC junction may be an effective DBS target for better symptom control (131).

Although the purpose of this article was to highlight neuromodulation, surgical lesioning strategies should be mentioned as well as there have been technological advances that have made this a safer and more precise strategy with comparable outcomes to DBS (158). Cingulotomies and capsulotomies are well-established techniques that have been improved upon over the past several decades, and multiple innovations such as stereotactic navigation, image-guidance, stereotactic radiosurgery, laser interstitial thermal therapy (LITT), and now MR-guided focused ultrasound (MRgFUS) have made lesioning a more viable option (159). Hageman et al. performed a meta-analysis to compare the outcomes for lesioning when compared to DBS for OCD (158). They found that there were equivalent responses and effect sizes. Both DBS and surgical lesioning come with the risk of hemorrhage, infection, suicidality, impulsivity, hypomania, and sleep changes. DBS had a statistically significant increased incidence of impulsivity when compared to ablation. Modern lesioning procedures do have some advantages over invasive neuromodulatory strategies (160). Potential advantages of DBS over lesioning relate to the reversibility of the procedure, and with lesioning, any targeting errors could lead to permanent off-target effects. Although there is no cost-effectiveness analysis to compare DBS vs. lesioning, the cost of the device, the visits to optimize parameters, and generator replacements certainly makes DBS more expensive and cumbersome than lesioning. Cost effectiveness analyses were performed in two international studies (161, 162), but there is a clear need to analyze the costs of DBS in the US. Other disadvantages of DBS relative to ablation is surgical incisions or modifiable device may become an object of obsession (163).

Overall, our opinion is that neuromodulatory strategies in the treatment of OCD are better than lesioning as new technologies will lead to more adaptable, patient-specific outcomes, and they do not involve permanent lesioning of critical brain structures.

Despite some of the limitations in DBS for OCD, it remains a promising strategy in the treatment of refractory OCD, and it continues to be an active area in research. Perhaps the most promising strategy to improve outcomes for a variety of pathologies is with closed-loop stimulation or adaptive stimulation (164). This would allow more adaptable, patient-specific, and symptom-appropriate stimulation. There are several clinical trials ongoing with the goal of demonstrating the efficacy of adaptive stimulation (NCT02773082, NCT03457675, NCT04281134, NCT04806516). Other active studies are looking provide more support for DBS to strial targets, such as ALIC/NAcc (NCT04967560), VIC and NAcc (NCT04228744), and VC/VS (NCT04217408). These ongoing clinical trials will hopefully help to define an optimal target and provide a groundwork for adaptive DBS.

## Conclusion

In summary, while early research into neural correlates of OCD has focused on individual components, several network-based models have emerged from more recent data on dysfunction within brain networks, including the lOFC-ventromedial caudate, limbic, salience, and default mode networks. Moreover, the interplay between multiple brain networks has been increasingly recognized. The implications of this for neuromodulation therapies in OCD is a shift more holistically toward modulation of networks. As our understanding of the neural circuitry underlying the pathophysiology of OCD continues to evolve, so will too our ability to specifically target these networks using invasive and non-invasive methods.

## Author contributions

AK created the manuscript and contributed to the DBS section. JC and JL contributed to the networks and TMS sections. AF and XM provided resources, guidance, and reviewed the manuscript. WC, MP, and KB contributed figures and edits to the manuscript. BV and SM provided useful feedback and edits to the manuscript. DL oversaw the project and outlined the review. All authors contributed to the article and approved the submitted version.

## Conflict of interest

The authors declare that the research was conducted in the absence of any commercial or financial relationships that could be construed as a potential conflict of interest.

## Publisher's note

All claims expressed in this article are solely those of the authors and do not necessarily represent those of their affiliated organizations, or those of the publisher, the editors and the reviewers. Any product that may be evaluated in this article, or claim that may be made by its manufacturer, is not guaranteed or endorsed by the publisher.
